# Surface Defect-Induced Dispersion and Stabilization of Monolayer MoS_2_ Nanosheets in Polar Solvents

**DOI:** 10.1021/cbe.4c00183

**Published:** 2025-05-16

**Authors:** Yuxin Zhang, Yishu Chen, Zhengqi Peng, Deliang Wang, Chengzhi Fu, Pingwei Liu

**Affiliations:** † State Key Laboratory of Chemical Engineering and Low-Carbon Technology, College of Chemical and Biological Engineering, 601555 Zhejiang University, Hangzhou 310027, P. R. China; ‡ Institute of Zhejiang University - Quzhou, 99 Zheda Road, Quzhou 324000, P. R. China

**Keywords:** dispersibility, monolayer MoS_2_, two-dimensional materials, surface free energy, surface defect

## Abstract

Effective dispersion of two-dimensional (2D) nanosheets in polar solvents is essential for their practical applications. However, ultrathin MoS_2_ nanosheets produced via mechanical exfoliation or liquid-phase exfoliation lack surface functionalities, posing a significant challenge for achieving a uniform dispersion and good colloidal stability. Here, we investigate the dispersion properties and stabilization mechanism of monolayer MoS_2_ colloids synthesized via a bottom-up strategy under nanoconfinement. The nanosheets achieve high dispersion concentrations of >1.6 g/L in polar solvents such as water, *N*-methylpyrrolidone, and 1,4-butanediol, with the highest concentration approaching 10.6 g/L in ethylene glycol, significantly higher than the previously reported concentrations of less than 0.8 g/L for the exfoliated MoS_2_ nanosheets. The surface free energy of our MoS_2_ nanosheets is determined to be 48.7 mJ/m^2^, from which their maximum stable dispersion concentrations in various solvents can be predicted precisely. The high surface free energy can be attributed to the presence of abundant surface defects on the nanosheets, which induce the formation of polar hydroxyl (−OH) groups and increase the negative charge density on the surface, thereby enhancing their dispersibility and colloidal stability. These findings hold significant implications for colloidal applications of 2D MoS_2_ nanosheets in various fields.

## Graphical abstract

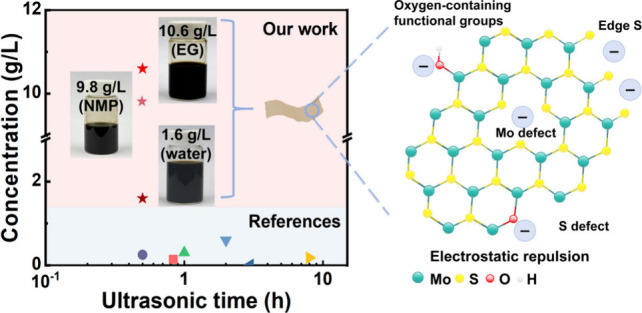

## Introduction

Single-layer transition metal dichalcogenides (TMDCs) are a promising class of two-dimensional materials renowned for their exceptional electronic,^1,2^ optical,^3,4^ and mechanical properties.^5^ Among the various TMDCs, MoS_2_ nanosheets stand out for their ease of synthesis, tunable electronic properties,^6^ and lubricating characteristics,^7–9^ further enhanced by their high surface area and active sites^10^ in monolayers. These attributes make them exceptionally versatile, enabling their use in a wide range of applications, from anodes in sodium-ion^11^ and lithium-ion^12,13^ batteries to surface coatings,^14^ polymer composites,^15–17^ catalysts,^18^ and beyond. In addition, MoS_2_ nanosheets are reported to be low-toxic and biocompatible^19–21^ and are widely used in heavy metal pollution remediation^22–25^ and biomedical^26^ fields. For these applications, it is highly necessary to form an optimal dispersion of MoS_2_ nanosheets with a high concentration, long-term stability, and uniform distribution without agglomeration.^27,28^ However, these ultrathin nanosheets, with their large surface area, are prone to agglomeration in solvents.^29^ In particular, MoS_2_ nanosheets prepared by mechanical^26,30–33^ or liquid-phase^34–37^ exfoliation often lack surface functional groups, making their direct dispersion in solvents highly challenging.

Dispersing MoS_2_ nanosheets at high concentrations typically involves enhancing surface charge^38^ or matching the surface free energy of the nanosheets with that of the solvent.^39^ Two primary strategies are commonly employed: (1) surface modification using functional groups (e.g., carboxyl,^40–42^ thiol,^43–48^ etc.^49–53^ ) and (2) surfactant-assisted dispersion with stabilizers such as polyvinylpyrrolidone,^54,55^ hexadecyltrimethyl­ammonium bromide,^56,57^ polyethylene glycol,^58–60^ and others.^61–63^ While surface modification enhances solvent interactions, it often introduces process complexity and chemical inhomogeneity. Surfactant-based methods offer a more straightforward approach but can lead to postprocessing challenges due to residual stabilizers.^64^ These limitations underscore the need for more efficient dispersion techniques to optimize MoS_2_ nanosheets for diverse applications.

Recent studies show that MoS_2_ nanosheets synthesized under nanoconfinement created by the interlayer space of layered double hydroxide (LDH)^65,66^ exhibit good dispersibility in polar solvents, even without the addition of stabilizing agents or surfactants. However, the precise mechanism by which these high concentrations are achieved remains unclear. In this study, we investigated the dispersion properties and stabilization mechanisms of the mentioned MoS_2_ nanosheets. We systematically evaluated their dispersions in various polar solvents, determining the maximum dispersion concentrations for each, with high dispersion concentrations exceeding 1.6 g/L in water and the highest concentration reaching 10.6 g/L in ethylene glycol. The surface free energies of MoS_2_ nanosheets were also calculated to predict their dispersion capabilities in different polar solvents. We further examined the structural characteristics and surface properties of the nanosheets using spectroscopic techniques and density functional theory (DFT) calculations to elucidate the unique dispersion mechanism. Our results indicate that surface defects play a crucial role in stabilizing the dispersions. This study underscores the potential of defect-induced stabilization as an innovative strategy to enhance the solvent dispersibility of MoS_2_ nanosheets.

## Materials and Experiment

### Materials

Al­(NO_3_)_3_·9H_2_O (AR, ≥99.0%), Mg­(NO_3_)_2_·6H_2_O (AR, ≥99.0%), hydrochloric acid (AR, 36.0–38.0%), ethanol (GR, ≥99.8%), (NH_4_)_2_MoS_4_ (AR, 99.0%), urea (AR, 99.0%), ethylene glycol (AR, ≥99.5%), 1,4-butanediol (GC, ≥99%), isopropyl alcohol (AR, ≥99.7%), methylbenzene (AR, ≥99.5%), *N*-methylpyrrolidone (AR, ≥99.5%), commercial bulky molybdenum disulfide (99.0%), and NaOH (AR, ≥96.0%) were purchased from Sinopharm Chemical Reagent Co., Ltd., China. Exfoliated MoS_2_ nanosheet was purchased from Xfnano Materials Tech Co., Ltd., China. All of the reagents were used without any further purification. Water was purified by using a water purification system (Canshi, China).

## Experimental Details

### Synthesis of Monolayer MoS_2_ Nanosheets

A nanoconfinement strategy previously reported in the literature^65^ was used to synthesize monolayer 2H-MoS_2_ nanosheets. First, a Mg–Al–CO_3_ LDH was synthesized hydrothermally.^67^ A solution of 7.5 g of Al­(NO_3_)_3_·9H_2_O (0.02 mol), 10.2 g of Mg­(NO_3_)_2_·6H_2_O (0.04 mol), and 27.7 g of urea (0.46 mol) in 300 mL of water was stirred and maintained at 90 °C for 24 h. LDH was obtained, which was then calcined at 400 °C for 2 h to form layered double oxide (LDO). For the MoS_4_^2–^ intercalated LDH (LDH-MoS_4_^2–^), 2.0 g of LDO and 3.0 g of (NH_4_)_2_MoS_4_ were reacted in 100 mL of water at room temperature for 24 h, producing the LDH-MoS_4_^2–^ composite. By calcining LDH-MoS_4_^2–^ at 500 °C for 2 h in N_2_, the LDO-MoS_2_ composite was obtained. To obtain monolayer MoS_2_, 4.0 g of LDO-MoS_2_ was dispersed in 100 mL of 0.5 M HCl, followed by 10 min of ultrasonication and 12 h of stirring. The solid was filtered and washed, yielding a gel-like MoS_2_ sample (48.4 wt %).

The 1T-MoS_2_ nanosheets were synthesized following a similar nanoconfined method with slight modifications.^66^ Mg–Al–CO_3_ LDH was first prepared using a hydrothermal process, then calcined at 450 °C for 2 h to produce LDO. The LDO was reacted with (NH_4_)_6_Mo_7_O_24_·4H_2_O, yielding the nanosized LDH-Mo_7_O_24_^6–^ precursor. This precursor was mixed with thiourea and ethanol and subjected to solvothermal treatment at 200 °C for 24 h, resulting in LDH-MoS_2_ powder. Monolayer 1T-MoS_2_ nanosheets were obtained by dissolving this powder in HCl, followed by ultrasonic processing and stirring.

Calculations indicate that raw materials (sulfur, molybdenum sources, and LDH) constitute approximately 80% of the synthesis cost, with calcination and acid washing accounting for the remaining 20%.

### Preparation of a MoS_2_ Nanosheet Dispersion

To prevent the aggregation of MoS_2_ nanosheets after drying, a centrifugation and solvent exchange strategy was employed. The gel-like MoS_2_ was directly dispersed in deionized water and a selection of polar solvents, including ethanol (EtOH), ethylene glycol (EG), 1,4-butanediol (BDO), isopropyl alcohol (IPA), and *N*-methylpyrrolidone (NMP), along with a gradient mixture of ethanol and deionized water (E/H, with ethanol volume fractions ranging from 0% to 100%). Additionally, methylbenzene (MB), a nonpolar solvent, was introduced to broaden the solvent scope. The dispersions were centrifuged at a relative centrifugal force (RCF) of 1000*g* for 10 min, followed by decanting of the supernatant to remove loosely bound impurities and excess solvent. This process was iteratively repeated 3 times for each solvent. The dispersions were sonicated in an ultrasonic bath for 0.5 h in sealed glass vials, achieving well-dispersed MoS_2_ nanosheets in the target solvents. Each dispersion was then analyzed by using ultraviolet–visible (UV–vis) absorption spectra to determine its concentration.

### Static Stability for the MoS_2_ Nanosheet Dispersion

The static stability of the MoS_2_ nanosheet dispersion was comprehensively evaluated by calculating the ratio (*c*_1_/*c*_0_), where *c*_1_ represents the concentration of the supernatant obtained after a 24 h settling period, and *c*_0_ is the initial dispersion concentration.^68^ For this purpose, dispersions of MoS_2_ nanosheets were prepared at a concentration of 1.0 g/L in various solvents, including water, EtOH, EG, BDO, IPA, and NMP, employing the aforementioned dispersion preparation protocol. Subsequently, after the dispersions were allowed to rest undisturbed for 24 h, the supernatant was carefully collected and analyzed using UV-spectrophotometry to determine its concentration (*c*_1_). This *c*_1_/*c*_0_ ratio served as a quantitative indicator of the dispersion stability of the MoS_2_ nanosheets in the respective solvents.

### Determination of the Maximum Dispersion Concentrations

The maximum dispersion concentration was evaluated via a centrifugation method.^69^ Taking the dispersion of nanosheets in water as an example, dispersions with initial concentrations (*c*_0_) ranging from 0.1 to 4.0 g/L were centrifuged at an RCF of 1000*g* for 10 min. The residual concentrations of MoS_2_ (*c*_2_) in the dispersions were determined, and *c*_2_ was plotted against *c*_0_. When the slope underwent a noticeable change, the corresponding *c*_2_ value was considered as the maximum dispersion concentration of MoS_2_ nanosheets in water. The maximum dispersion concentrations in EtOH, EG, BDO, IPA, and NMP were investigated using the same centrifugation method.

### Surface Free Energy of MoS_2_ Nanosheets

The surface free energy components of MoS_2_ could be calculated according to Young’s equation and the OWRK method (Owens, Wendt, Rabel, and Kaelble model) from the contributions of the solvent and the solid by forming a geometric mean based on eq 1 with the acquired contact angles.^70^ The surface free energy of MoS_2_ nanosheets (*γ*_*s*_) is determined according to eq 2.

1 $$0.5\left(1+\cos\theta \right)\gamma_{L}/{\left(\gamma_{d,L}\right)}^{1/2}={\left(\gamma_{p,S}\right)}^{1/2}\cdot {\left(\gamma_{p,L}/\gamma_{d,L}\right)}^{1/2}+{\left(\gamma_{d,S}\right)}^{1/2}$$

2 $$\gamma_{s}=\gamma_{p,s}+\gamma_{d,s}$$

Here, *θ* is the contact angle of the solvent with different surface free energy on the MoS_2_ nanosheet surface, *γ*_*L*_ is the total surface free energy of the solvent, *γ*_*d,L*_ is the dispersive component of the solvent surface free energy, *γ*_*p,L*_ is the polar component of the solvent surface free energy, *γ*_*S*_ is the total surface free energy of MoS_2_ nanosheets, *γ*_*p,S*_ is the polar component of the MoS_2_ nanosheet surface free energy, and *γ*_*d,S*_ is the dispersive component of the MoS_2_ nanosheet surface free energy.

Five solvents with different surface free energy components, including deionized water, EG, NMP, EtOH, and IPA were chosen to measure the surface free energy of MoS_2_ nanosheets. The values for the polar component *γ*_*p,L*_, the dispersive component *γ*_*p,L*_, the total surface free energy *γ*_*L*_, and the contact angle for the five solvents are shown in Table S1.

## Characterization

Atomic force microscope (AFM) images were acquired on a Multimode SPM (VEECO, USA) and a nanoIR2 fs (Anasys Instruments, USA) in tapping mode using a Si-tip cantilever, and the test sample was deposited on a mica wafer. Scanning electron microscopy (SEM) and energy-dispersive X-ray spectroscopy (EDS) mapping measurements were carried out using an SU8010 microscope (Hitachi, Japan). Transmission electron microscopy (TEM) images were collected on an HT-7700 Hitachi TEM system (Hitachi, Japan) operating at 120 kV. The sample was ultrasonically dispersed in water (0.01 g/L) for 10 min, and then a drop of the dispersion was dropped on a copper TEM grid. The ultraviolet–visible (UV–vis) absorption spectra were determined by using a TU-1901 spectrophotometer (PERSEE, China).

The size distribution and zeta potential of particles were analyzed using dynamic light scattering (DLS) (Zetasizer Nano-ZS90, Malvern, England). The size distribution and visual observation of the MoS_2_ nanosheets in the dispersions were characterized by using a nanoparticle tracking analysis (NTA) system (Zetasizer Nano-ZS, Malvern, England). Samples were diluted to 10^8^ particles/mL in deionized water to achieve an optimal particle concentration for analysis. Rheological tests with MoS_2_ dispersions (2.5–20.0 g/L) were conducted using a rotational rheometer (MARS 60, HAAKE, Germany) at shear rates ranging between 0.1 s^–1^ and 100 s^–1^ with a double-gap rheometer setup (amount: 4.5 mL) due to apparent low viscosity. Contact angle measurements were performed using a video-based contact angle measuring device (OCA 20, DataPhysics Instruments, Germany).

X-ray photoelectron spectroscopy (XPS) spectra were recorded on a 250Xi spectrometer (Thermo Fisher, USA). The 1s C peak at 284.6 eV was used to calibrate the XPS peak positions. X-band electron paramagnetic resonance (EPR) spectra were obtained by using a Bruker EMX spectrometer (Bruker, Germany).

The present first-principles DFT calculations are performed by the Vienna Ab initio Simulation Package (VASP) with the projector augmented wave (PAW) method. The exchange-functional is treated using the generalized gradient approximation (GGA) of the Perdew–Burke–Emzerhof (PBE) functional. The energy cutoff for the plane wave basis expansion was set to 450 eV, and the force on each atom less than 0.05 eV/Å was set for convergence criterion of geometry relaxation. Grimme’s DFT-D3 methodology was used to describe the dispersion interactions. Partial occupancies of the Kohn–Sham orbitals were allowed using the Gaussian smearing method and a width of 0.05 eV. The Brillouin zone was sampled with a 3 × 3 × 1 Monkhorst mesh through all of the computational processes. The self-consistent calculations apply a convergence energy threshold of 10^–5^ eV.

## Results and Discussion

The monolayer 2H phase MoS_2_ (M-2H-MoS_2_) nanosheet sample synthesized via a nanoconfinement method^66^ was first characterized with different microscopy techniques. Figure 1A shows the AFM image of the M-2H-MoS_2_. Their average thickness and lateral size based on statistics of ∼400 particles are ∼1.0 and 200 nm (Figure 1B and C), respectively. The monolayer ratio of M-2H-MoS_2_ nanosheets is determined to be ∼96% (Figure 1C). SEM showed that MoS_2_ nanosheets present an irregular flaky morphology (Figure S1A). TEM revealed the ultrathin nature of these M-2H-MoS_2_ nanosheets with quite a weak contrast (Figure S1B). The UV–vis absorption spectra displayed three prominent peaks at wavelengths (λ) of 420, 610, and 661 nm (Figure S2A), which are attributed to the 2H semiconductor phase of MoS_2_. The absorbance at 661 nm showed a strong correlation with concentration, consistent with the Lambert–Beer law (Figure S2B). The absorption coefficient (α) was determined to be approximately 1317 L g^–1^ m^–1^, which aligns well with literature-reported values for monolayer 2H-MoS_2_ dispersions.^69^ Additionally, exfoliated MoS_2_ was also characterized, revealing nanosheets with heterogeneous sizes and a mixed crystal structure (Figure S3).

**Figure 1:**
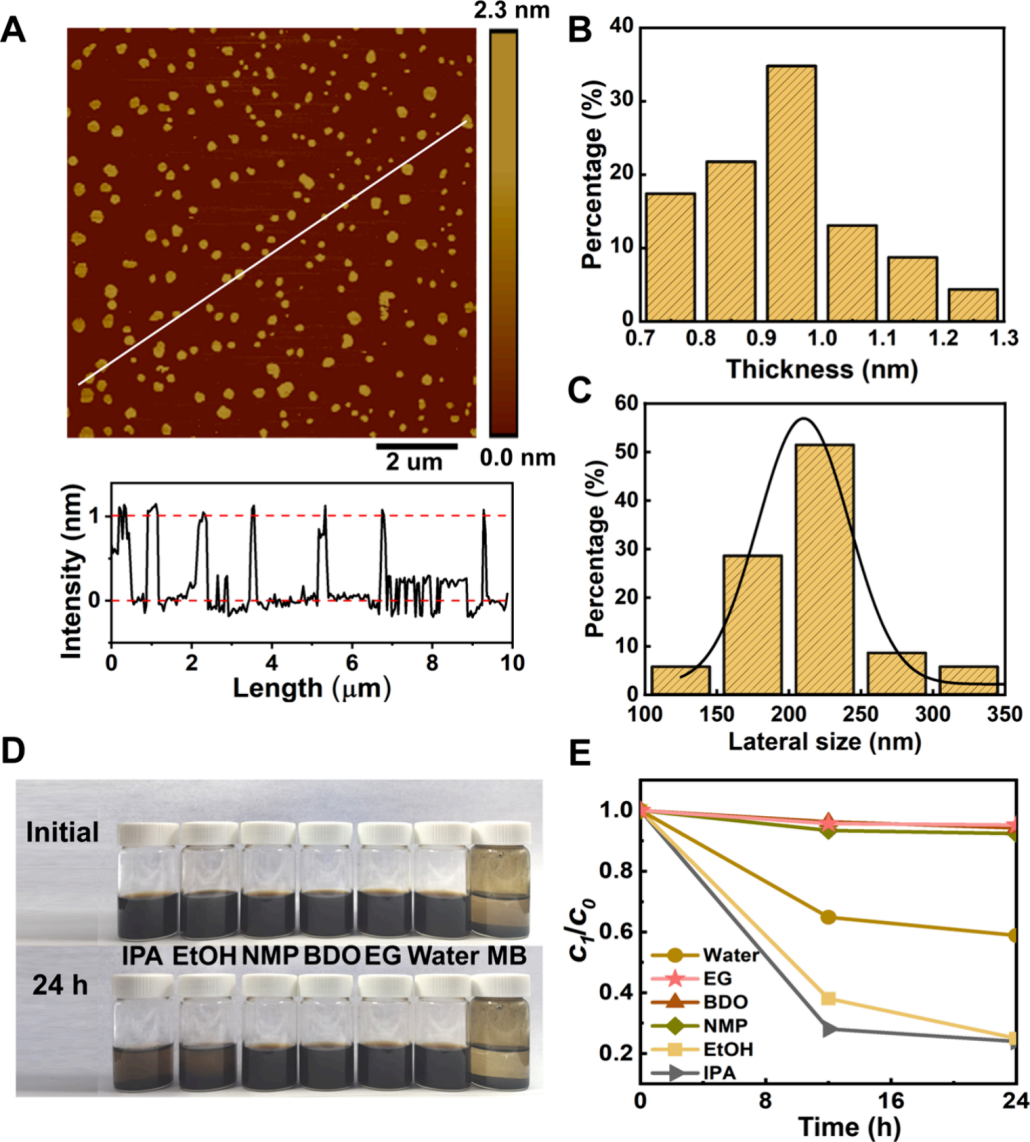
Characterization of M-2H-MoS_2_ nanosheets and dispersions. (A) AFM images of M-2H-MoS_2_ nanosheets. (B) Thickness and (C) lateral size distributions. (D) Photograph of M-2H-MoS_2_ nanosheet dispersions in various solvents after 24 h of static sedimentation. (E) Static stability for M-2H-MoS_2_ nanosheet dispersions (*c*_1_/*c*_0_) as a function of static placing time in different polar solvents.

We investigated the dispersion stability of M-2H-MoS_2_ nanosheets by analyzing their dispersions in various solvents after 24 h of static sedimentation. Upon sonication, M-2H-MoS_2_ nanosheets could be well dispersed in all six polar solvents including IPA, EtOH, NMP, BDO, EG, and deionized water, whereas in the nonpolar solvent, MB, M-2H-MoS_2_ nanosheets could not be dispersed (Figure 1D). To quantify this stability, the ratio of the postsedimentation concentration of supernatant (*c*_1_) to its initial concentration (*c*_0_) was calculated. As shown in Figure 1E, M-2H-MoS_2_ dispersions in EG, BDO, and NMP exhibit high stability, with *c*_1_/*c*_0_ values larger than 90.0%, which is better than 58.9% in water. Furthermore, the MoS_2_ dispersion in EG shows no noticeable sedimentation after 7 days (Figure S4), indicating a good dispersion stability. This long-term stability is superior to that reported in prior studies (Table S2).

The dispersibility of M-2H-MoS_2_ nanosheets in water was systematically investigated. In a diluted M-2H-MoS_2_ dispersion (0.02 g/L), the Tyndall effect, arising from light scattering by M-2H-MoS_2_ nanosheets, was clearly observable (inset, Figure 2A). DLS tests reveal that M-2H-MoS_2_ colloids are pH-sensitive and exhibit reducing zeta potential values from −28.1 mV to −43.6 mV with rising pH from 3.0 to 11.0, respectively (Figure 2A). Particularly in the neutral and alkaline conditions, their absolute values are all larger than 30 mV, indicating a good dispersion.^71^ This pH-dependent charge variation arises from interactions between nanosheet surface groups and H^+^ or OH^–^ ions. In acidic media, excess H^+^ ions partially neutralize the negative charges on the nanosheet, particularly at sulfur sites and hydroxyl groups along the edges, reducing the absolute zeta potential. In alkaline conditions, OH^–^ ions increase the inherent negative charge density of the nanosheets, enhancing electrostatic repulsion between them. Correspondingly, the Z-average diameter exhibited a pH-dependent reduction, initially averaging ∼290 nm at pH 3.0 and gradually decreasing to stabilize around 130 nm above pH 7.0. The NTA results show that at pH below 7.0 a broad particle size distribution was observed, with a notable presence of agglomerates exceeding 300 nm (Figure S5). As the pH was adjusted from 7.0 to 11.0, the number-average diameter remained stable in the range 150.3–154.9 nm, consistent with DLS results.

**Figure 2:**
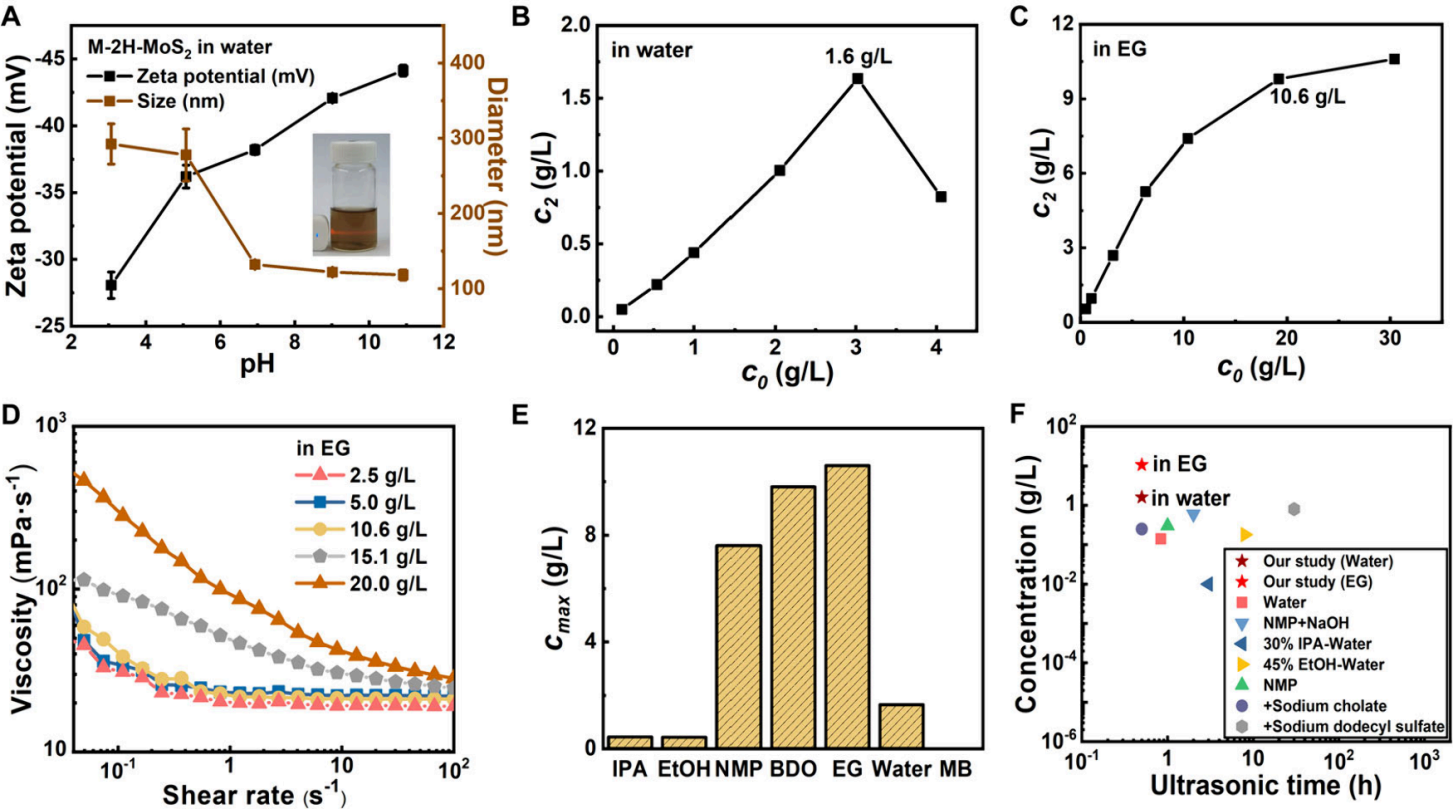
Dispersion stability of M-2H-MoS_2_ nanosheets. (A) Zeta potentials (black) and Z-average diameter (brown) of M-2H-MoS_2_ as a function of pH values. (B) Residual dispersion concentration (*c*_2_) as a function of initial M-2H-MoS_2_ concentration (*c*_0_) in water. (C) *c*_2_ as a function of *c*_0_ in EG. (D) M-2H-MoS_2_ dispersion viscosity as a function of the shear rate in EG. (E) Maximum dispersion concentration (*c*_*max*_) of M-2H-MoS_2_ in seven different solvents. (F) Comparison of the dispersion concentration of MoS_2_ dispersions with different methods.

The dispersion stability of M-2H-MoS_2_ nanosheets in water at a pH of 7.0 was further investigated. Figure 2B illustrates the change in the residual concentrations (*c*_2_) after centrifugation with different initial concentrations (*c*_0_) for an M-2H-MoS_2_ dispersion in water. As *c*_0_ increases from 0.1 to 4.0 g/L, *c*_2_ initially increases, peaking at 1.6 g/L around *c*_0_ ∼ 3.0 g/L, before decreasing. The maximum stable concentration^69^ of M-2H-MoS_2_ nanosheets (*c*_*max*_) in water is thus determined to be 1.6 g/L.

We further studied the dispersibility of M-2H-MoS_2_ nanosheets in EG. As *c*_0_ increases from 0.5 to 30.4 g/L, *c*_2_ continuously increases until the slope decreases slightly after *c*_2_ exceeds 10.6 g/L (Figure 2C), suggestive of aggregation occurring at concentrations exceeding this threshold. Rheological analysis further confirmed that at concentrations over 10.6 g/L a sharp viscosity drop at high shear rates indicates the aggregation^72^ of M-2H-MoS_2_ nanosheets in EG (Figure 2D), aligning with prior results. Besides, the *c*_*max*_ values of MoS_2_ nanosheets in IPA, EtOH, BDO, and NMP were determined to be 0.4, 0.4, 7.6, and 9.8 g/L, respectively (Figure 2E). Notably, *c*_*max*_ of M-2H-MoS_2_ in water and EG were significantly higher than the previously reported values^11,18,35,73–79^ (i.e., less than 0.8 g/L) for the exfoliated MoS_2_ nanosheets dispersed in diverse solvents, even including those with surfactant assistance, as summarized in Figure 2F.

We further characterized the surface free energy of M-2H-MoS_2_ nanosheets to understand their dispersion behavior in different solvents. Initially, the contact angles of a range of solvents on the M-2H-MoS_2_ surface were measured (inset, Figure 3A). The water contact angle is 49.3° for M-2H-MoS_2_ nanosheets and 71.1° for bulky MoS_2_ (see Figure S6), indicating a higher hydrophilicity of these M-2H-MoS_2_ nanosheets. Young’s equation and the OWRK theory were used to fit the experimental data (Figure 3A), and the surface free energy of M-2H-MoS_2_ nanosheets was calculated to be 48.7 mJ/m^2^ (see the details in the Experimental Section), which is notably higher than the previously reported values for conventionally exfoliated MoS_2_ nanosheets (less than 40 mJ/m^2^).^70,80^

**Figure 3:**
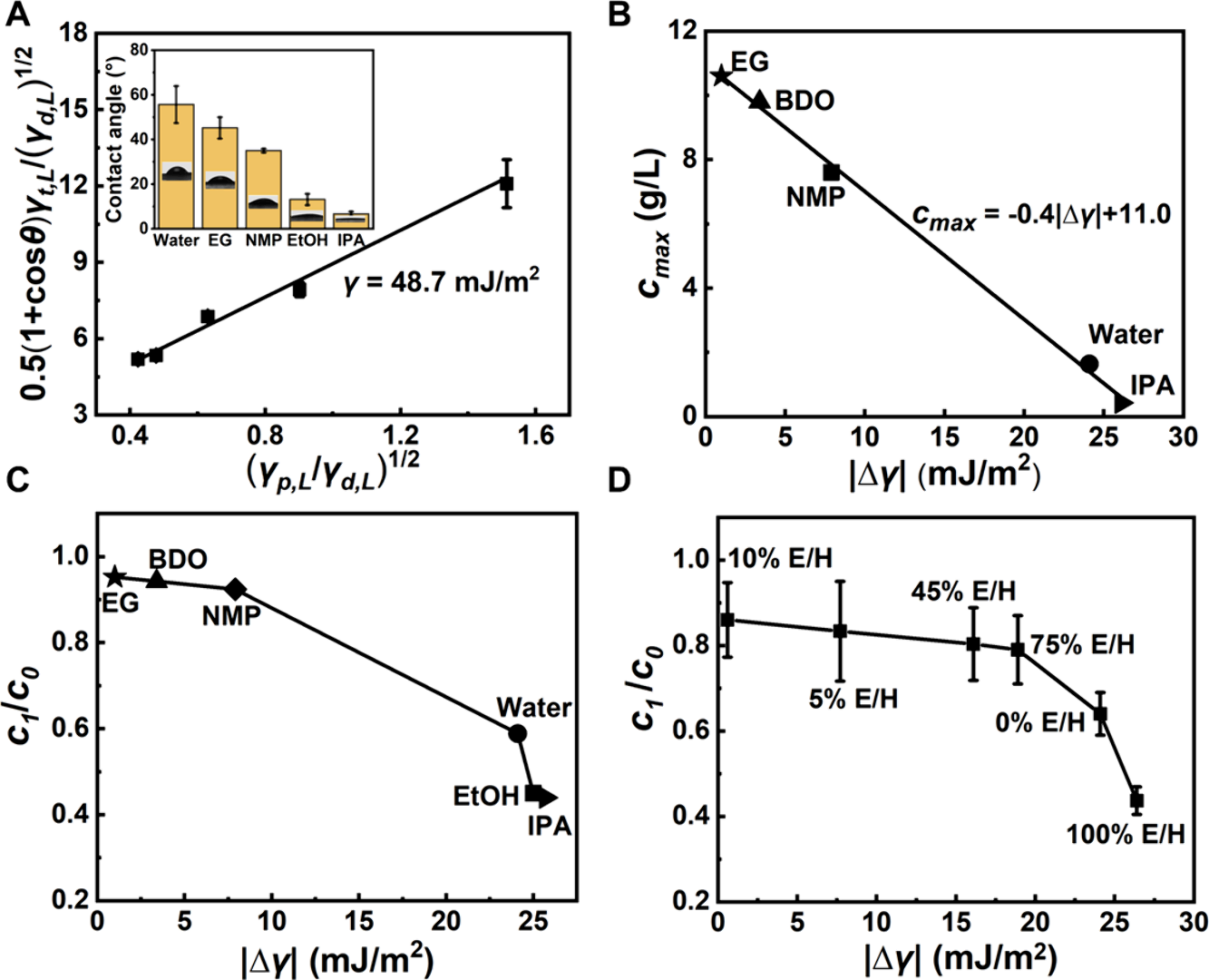
Effect of surface free energy on dispersion. (A) Linear fit of the OWRK equation with various solvents to obtain surface free energy (*γ*) of M-2H-MoS_2_ nanosheets. (Inset: contact angles of nanosheet film with five solvents.) (B) Linear fit of maximum stable dispersion concentration (*c*_*max*_) to the difference between the surface free energy of M-2H-MoS_2_ nanosheets and the solvents (|Δ*γ*|). (C) Static stability for M-2H-MoS_2_ nanosheet dispersion (*c*_1_/*c*_0_) as a function of |Δ*γ*|. (D) The effect of |Δ*γ*| on the dispersion stability was ascribed to adjusting the volume ratio of ethanol and water.

We found that the maximum stable dispersion concentration (*c*_*max*_) increases from 0.4 to 10.6 g/L as the solvent’s surface free energy approached that of the nanosheets. Since M-2H-MoS_2_ nanosheets are ultrathin, the effect of gravity on their dispersion could be neglected. Thus, matching the surface free energies of these nanosheets with the solvent becomes more critical for dispersion. By calculating the surface free energy absolute difference between the solvents and the nanosheets (|Δ*γ*| = |*γ*_*s*_ – *γ*_L_|), a linear relationship was discovered between |Δ*γ*| and *c*_*max*_ (Figure 3B). Based on this, the highest dispersion concentration of the nanosheets in the polar solvent was predicted to be 11.4 g/L, when the solvent’s surface free energy aligns with 48.7 mJ/m^2^. This finding facilitates the prediction of M-2H-MoS_2_ nanosheet dispersibility across diverse polar solvents.

To assess the dispersion stability of M-2H-MoS_2_ nanosheets in various solvents, we analyzed the ratio of supernatant concentration post-24-h standing to the initial dispersion concentration (*c*_1_/*c*_0_) as a function of |Δ*γ*|, as presented in Figure 3C. Notably, M-2H-MoS_2_ nanosheets exhibited the highest stability in EG, retaining 95.2% of their initial concentration, indicating that solvents with surface free energies closer to that of MoS_2_ nanosheets foster superior dispersion stability.

The correlation between |Δ*γ*| and M-2H-MoS_2_ dispersion stability was further confirmed through experiments conducted with mixed E/H solutions at varying ethanol volume fractions, ranging from 0% to 100% (Figure 3D, the surface free energies of these mixtures are detailed in Table S3). In particular, at 10% E/H with a surface energy of 48.1 mJ/m^2^, an optimal dispersion stability of M-2H-MoS_2_ nanosheets could be achieved.

We also considered the influence of the crystal phase of the MoS_2_ nanosheets. Similar to the above 2H phase, the surface free energy plays a critical role in the dispersion stability of monolayer 1T phase MoS_2_ (M-1T-MoS_2_) nanosheets. These nanosheets were synthesized via a nanoconfined solvothermal method^67^ (see their XRD result in Figure S7 and UV–vis spectroscopy in Figure S2A). The surface free energy of M-1T-MoS_2_ was determined to be 55.3 mJ/m^2^ (Figure S8A). When dispersed in EG, BDO, and NMP with the surface free energy close to this value, the nanosheets also exhibited high stability; that is, the dispersions did not show noticeable sedimentation after 24 h (Figure S8B). These results further highlight the crucial role of surface free energy in achieving the dispersion stability of MoS_2_ nanosheets.

The structural characteristics of M-2H-MoS_2_ nanosheets were studied by XPS to understand their dispersion properties. For comparison, the spectra of bulky MoS_2_ (B-MoS_2_, 2H phase) were also included as a control. Specifically, the Mo 3d_5/2_ and Mo 3d_3/2_ peaks for M-2H-MoS_2_ appear at binding energies (*E*_*B*_) of 229.6 and 232.8 eV (Figure 4A), corresponding to Mo^4+^. These peaks have a shift of 0.3 eV to higher *E*_*B*_ compared to B-MoS_2_, which can be attributed to the partial oxidation of Mo^4+^. This is supported by the observation of two weak peaks^81^ at an *E*_*B*_ of ∼233.1 and 235.7 eV, which can be assigned to Mo^6+^. The presence of Mo–OH and Mo–O bonds could be also directly observed at 533.5 and 530.6 eV in the O 1s spectrum (Figure 4B), respectively. These results confirm the surface oxidation of the M-2H-MoS_2_ nanosheets. In the S 2p spectra (Figure 4C), the S^2–^ peaks of M-2H-MoS_2_ nanosheets appear at an *E*_*B*_ of 162.4 eV (2p_3/2_) and 163.6 eV (2p_1/2_), 0.3 eV higher than that of the B-MoS_2_. This should be attributed to the oxidation of Mo^4+^ into Mo^6+^. The existence of vacancies was confirmed through magnetic field measurements using X-band EPR spectra (Figure 4D). The signature of *g* at 2.003 belonged to S vacancies^82^ in M-2H-MoS_2_, and another clear signal around 1.94 was due to the presence^83^ of unpaired Mo^4+^. The presence of sulfur vacancies can lead to oxygen substitution, thus resulting in the partial oxidation of Mo^4+^. Therefore, the characteristic surface structure of M-2H-MoS_2_ with S vacancies, O substitution, and hydroxyl (−OH) groups explains its good dispersibility in various polar solvents.

**Figure 4:**
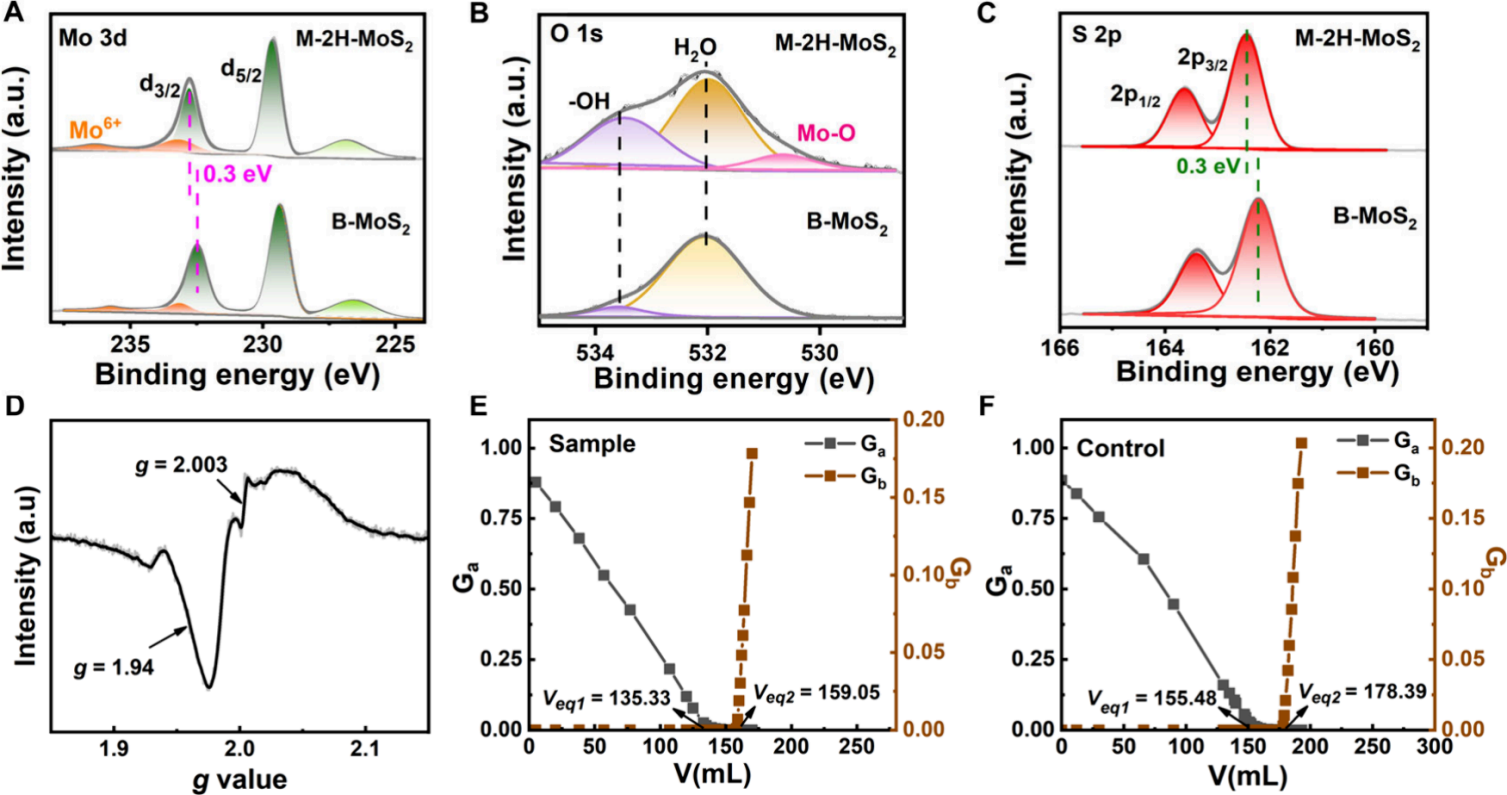
Structure characteristics of the M-2H-MoS_2_ nanosheets. XPS spectra of (A) Mo 3d, (B) O 1s, and (C) S 2p of M-2H-MoS_2_ and B-MoS_2_. (D) EPR spectra of M-2H-MoS_2_. Gran’s function plots for (E) sample (M-2H-MoS_2_ dispersion in water at 1.0 g/L) and (F) control (the supernatant of dispersion after centrifugation at 8000 rpm for 10 min).

An acid–base titration method in conjunction with Gran’s function analysis was further employed to determine the effective surface hydroxyl density on M-2H-MoS_2_ nanosheets.^84^ The Gran’s function plots for the M-2H-MoS_2_ nanosheet dispersion and its supernatant after centrifugation were measured (Figure 4E and 4F; see detailed experimental procedures and calculations in Section S1). The effective density of hydroxyl groups on M-2H-MoS_2_ nanosheets was determined to be 1.36 × 10^–5^ mmol/m^2^. Note that this value is only 1 order of magnitude lower than 1.55 × 10^–4^ mmol/m^2^ of the hydroxyl-rich LDHs with good water dispersibility.^85^ This explains the enhanced surface potential and good dispersibility of these M-2H-MoS_2_ nanosheets within various polar solvents.

We further conducted DFT calculations of the two-dimensional potential energy surface (see the structure representation of the 5 × 5 supercell of M-2H-MoS_2_ with defects in Figure 5A). The minimum-energy configuration of MoS_2_ corresponds to a situation with maximum overlap of unlike atoms: S over Mo.^86^ The introduction of vacancies induces three defect states: two filled states and one unfilled state. The substitution of sulfur (S) with oxygen (O) and the presence of edge hydroxyl groups both appear red, indicating a negatively charged density associated with these defect states (Figure 5B). Moreover, the presence of molybdenum (Mo) defects and edge sulfur (S) atoms further enhances the negative potential. The calculated total density of states (TDOS) reveals a nonzero density of states at the Fermi level (Figure 5C). The additional electronic states suggest that its electronic structure has been altered, indicating the introduction of negative charge. These findings indicate that defects create a negative surface potential, thus playing a crucial role in promoting the dispersion and stabilization of M-2H-MoS_2_ nanosheets in polar solvents.

**Figure 5:**
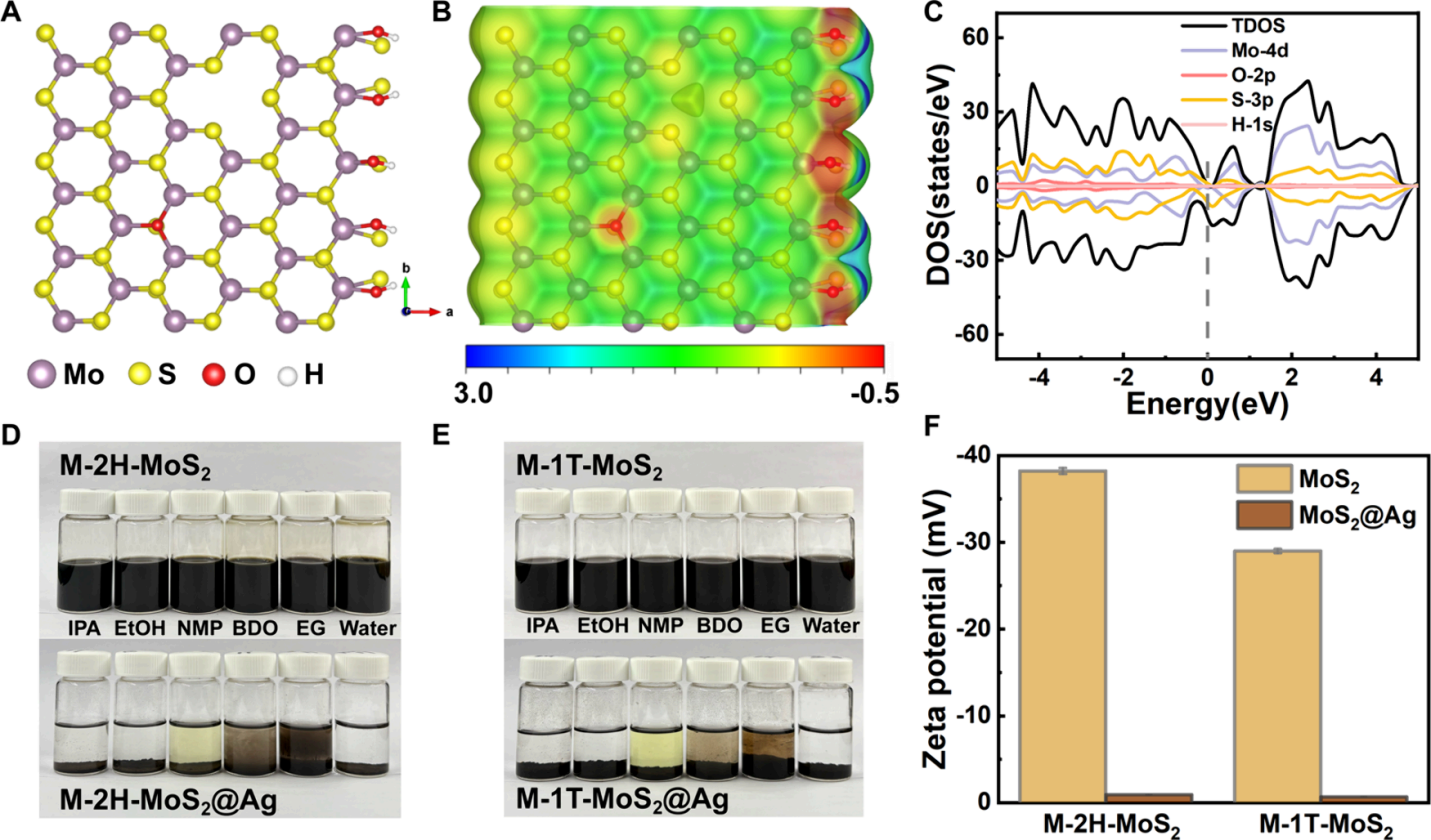
DFT calculation of monolayer MoS_2_ nanosheets and their interaction with Ag^+^. (A) Structure representation of M-2H-MoS_2_, computed via DFT in a 5 × 5 supercell. (B) The calculated charge density distribution of the M-2H-MoS_2_ surface. (C) The calculated total density of states (TDOS) and partial density of states (PDOS) for Mo, O, S, and H atoms. Photographs for the dispersions of (D) M-2H-MoS_2_ and (E) M-1T-MoS_2_ nanosheets in various polar solvents before and after adding Ag^+^ ions (34.0 mg AgNO_3_, i.e., 0.02 M Ag^+^). (F) Comparison of the zeta potential of MoS_2_ nanosheets before and after adding Ag^+^ ions.

To further confirm the negatively charged nature of monolayer MoS_2_ nanosheets rendering better dispersibility, metal ions were introduced for neutralization. Given the strong affinity of Ag^+^ ions to sulfur, as indicated by the extremely low solubility product constant (*K*_*sp*_) of Ag_2_S (1.6 × 10^–49^), Ag^+^ ion was selected to bind with the negatively charged unsaturated S sites. After addition of AgNO_3_ (34.0 mg, i.e., 0.02 M Ag^+^), a quick sedimentation of the nanosheets in polar solvents could be observed for both M-2H-MoS_2_ and M-1T-MoS_2_ (Figures 5D and 5E). Additionally, a remarkable reduction of the zeta potentials could be identified (Figure 5F): from −38.1 ± 0.3 mV to below −0.9 mV for M-2H-MoS_2_ and from −29.0 ± 0.3 mV to below −0.7 mV for M-1T-MoS_2_. The results highlight the critical role of unsaturated S with negative charges for dispersion. It suggests the application potential of these defective MoS_2_ nanosheets in the field of environmental remediation, e.g., for the removal of heavy metal ions.^22–25^

## Conclusions

In this study, we thoroughly investigated the dispersion characteristics of monolayer MoS_2_ nanosheets in various polar solvents. We achieved exceptionally high dispersion concentrations, reaching 10.6 g/L in ethylene glycol, which significantly exceeds the previously reported values of less than 0.8 g/L for exfoliated MoS_2_ nanosheets. The surface free energy was determined to be 48.7 mJ/m^2^ and 55.3 mJ/m^2^ for the 2H-phase and 1T-phase monolayer MoS_2_ nanosheets, respectively, synthesized via the nanoconfined method. Consequently, their dispersibility across different solvents can be accurately predicted. This impressive dispersibility and high surface free energy can be attributed to the nanosheets’ unique surface structure, featuring abundant sulfur vacancies and oxygen substitution, leading to the formation of hydroxyl groups and increased negative surface charge density. However, several limitations should be noted: (1) Environmental conditions can affect material dispersibility by influencing the functional groups and surface charge on nanosheet surfaces. (2) This method is particularly effective for ultrathin nanosheets, as they are less affected by gravity. Our findings underscore the potential for defect-induced dispersion and stabilization of monolayer MoS_2_ nanosheets in polar solvents, providing valuable insights for their colloidal applications in various fields.

## Supplementary Material



## Notes

Nanosheet characterizations including scanning electron microscopy, transmission electron microscopy, element mapping, energy dispersive X-ray spectroscopy results, UV–vis spectroscopy, Lambert–Beer plot, nanoparticle tracking analysis, X-ray photoelectron spectroscopy, contact angle images, and X-ray diffraction patterns; linear fit of OWRK equation with various solvents to obtain surface free energy of M-1T-MoS_2_ nanosheets and photograph of M-1T-MoS_2_ nanosheet dispersions in various solvents after 24 h of static sedimentation; raw materials and cost estimation; total surface free energy and surface free energy components of the solvents; determination of the effective surface hydroxyl density on M-2H-MoS_2_ nanosheets (PDF)
