# Supplementary material for: Surface Defect-Induced Dispersion and Stabilization of Monolayer MoS2 Nanosheets in Polar Solvents
Source: Chem Bio Eng. 2025 May 16;2(6):370–9. doi: 10.1021/cbe.4c00183 (PMC12207278; doi:10.1021/cbe.4c00183)
Supplement: Supplementary file 1 [file be4c00183_si_001.pdf]

Supplementary Information (SI):

Surface Defect-Induced Dispersion and Stabilization of Monolayer MoS_2 Nanosheets in Polar Solvents

Yuxin Zhang[a], Yishu Chen[a], Zhengqi Peng[a], Deliang Wang[a, b]*, Chengzhi Fu[a], Pingwei Liu[a, b]*

[a.] State Key Laboratory of Chemical Engineering and Low-Carbon Technology, College of Chemical and Biological Engineering, Zhejiang University, Hangzhou 310027, P. R. China

[b.] Institute of Zhejiang University - Quzhou, 99 Zheda Road, Quzhou 324000, P.R. China

E-mail: liupingwei@zju.edu.cn (Pingwei Liu)
E-mail: dlwang1991@zju.edu.cn (Deliang Wang)

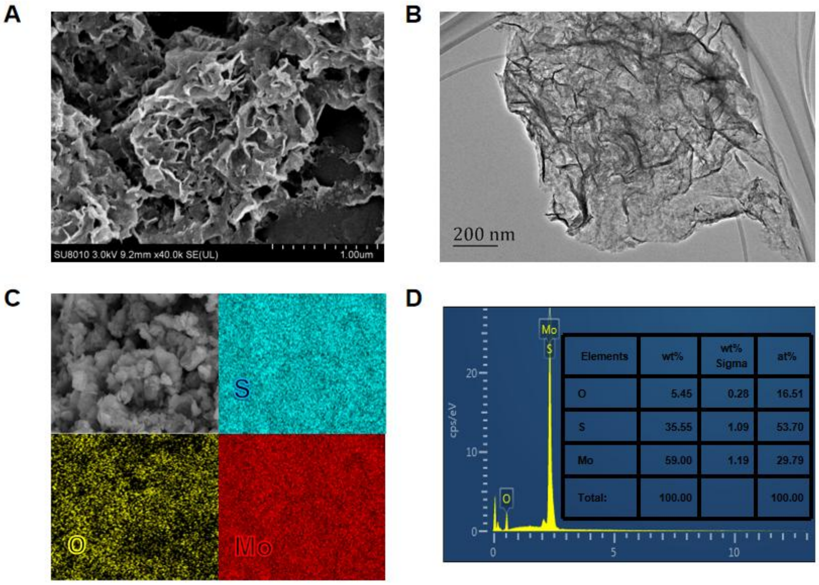

Figure S1. (A) SEM and (B) TEM images of M-2H-MoS_2 nanosheets. (C) Element mapping and (D) energy dispersive X-ray spectroscopy (EDS) results of M-2H-MoS_2 nanosheets.

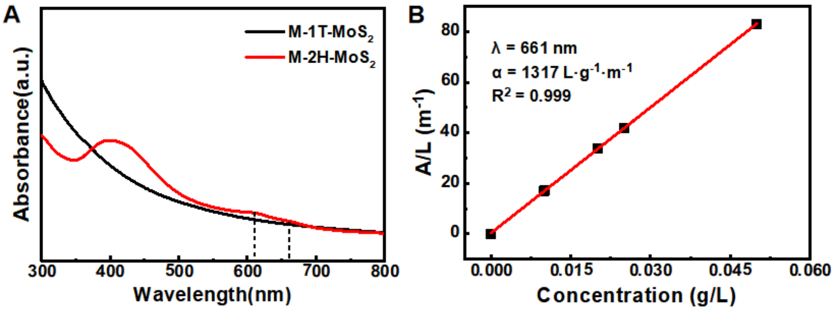

Figure S2. (A) UV-vis spectroscopy of M-1T-MoS_2 and M-2H-MoS_2. (B) Lambert-Beer plot of M-2H-MoS_2 dispersions at 661 nm.

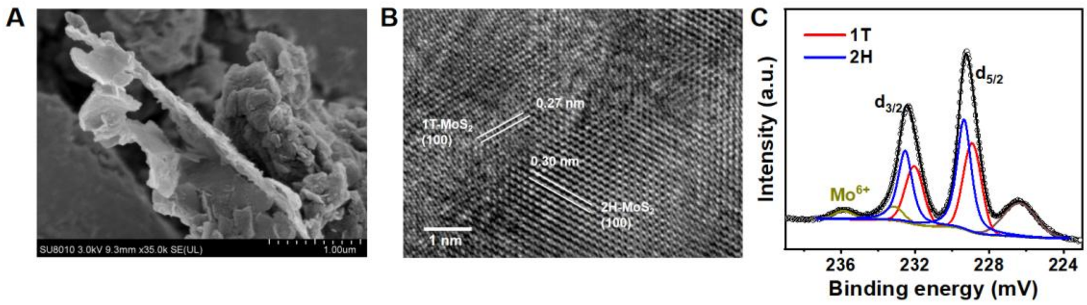

Figure S3. (A) SEM, (B) HRTEM images and (C) XPS spectra of exfoliated MoS_2 nanosheets.

The SEM image (Figure S3A) reveals that the exfoliated MoS_2 nanosheets exhibit a flaky morphology with heterogeneous sizes. The HRTEM image further illustrates the coexistence of two nanocrystals: the 1T-MoS_2 phase, characterized by a (100) inter-planar spacing of 0.27 nm, and the 2H-MoS_2 phase, with a (100) inter-planar spacing of 0.30 nm. The Mo 3d XPS spectrum (Figure S3C) was deconvoluted into two crystalline doublets, corresponding to 1T-MoS_2 and 2H-MoS_2. Additionally, due to the oxidation of the nanosheets, a peak associated with Mo^{6+} was observed.

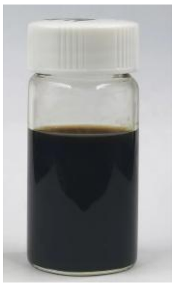

Figure S4. Photograph of the 1.0 g/L MoS_2 nanosheet dispersion in EG after 7 days under real-world conditions.

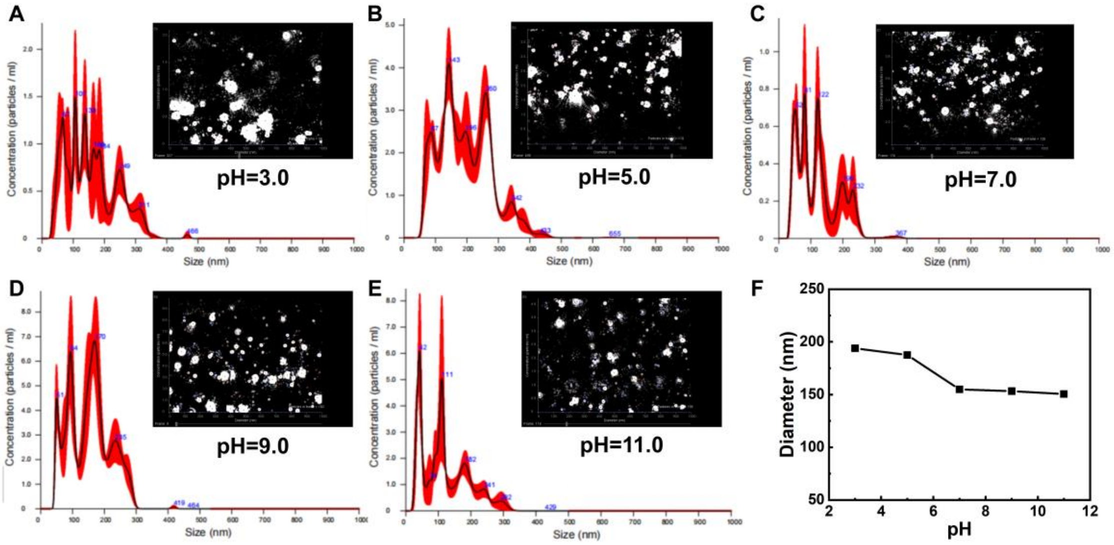

Figure S5. NTA with representative live stream views of M-2H-MoS_2 dispersions in (A) pH=3.0, (B) pH=5.0, (C) pH=7.0, (D) pH=9.0 and (E) pH=11.0. (F) The number-average diameter of M-2H-MoS_2 as a function of pH values.

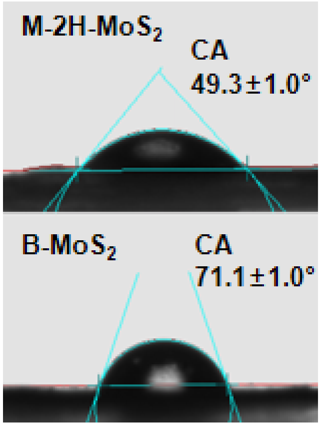

Figure S6. Contact angle images of M-2H-MoS_2 and B-MoS_2.

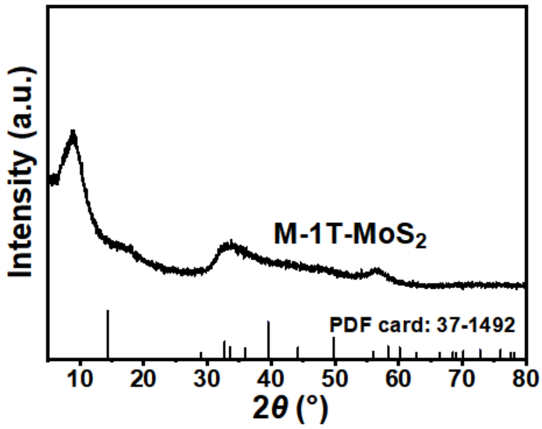

Figure S7. XRD patterns of M-1T-MoS_2.

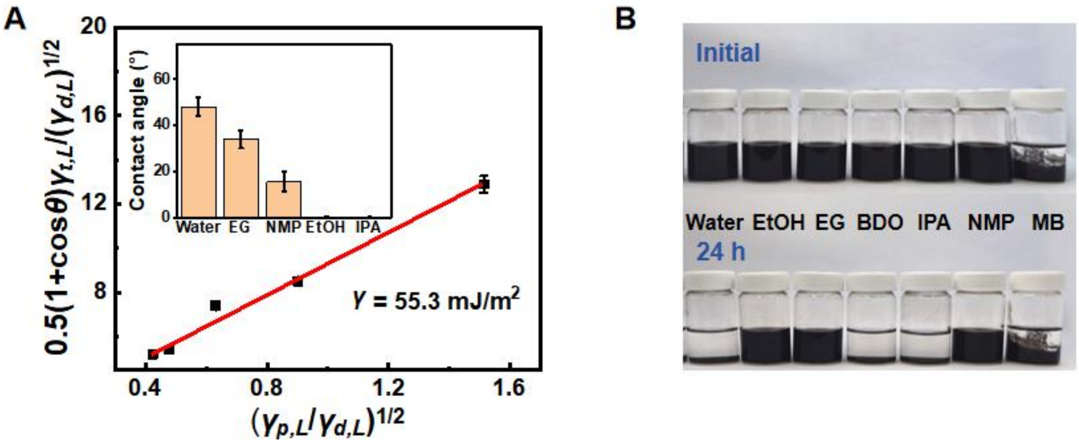

Figure S8. (A) Linear fit of OWRK equation with various solvents to obtain surface free energy (γ) of M-1T-MoS_2 nanosheets. (Inset: contact angles of nanosheets film with five solvents) (B) Photograph of M-1T-MoS_2 nanosheets dispersions in various solvents after 24 h of static sedimentation.

Table S1. Total surface free energy and surface free energy components of the solvents[1]

Solvents	γ_L (mJ/m^2)	$\gamma_{p,L}$ (mJ/m^2)	$\gamma_{d,L}$ (mJ/m^2)
Water	72.8	50.7	22.1
EG	47.7	21.4	26.3
NMP	40.8	11.6	29.2
EtOH	23.7	4.4	19.3
IPA	23.0	3.5	19.5
BDO	39.6	/	/

Table S2. Literature comparison of long-term stability

Method	Solvent	Aggregation time
[2]Exfoliation	Water	7 days
[3]Exfoliation	EtOH/Water	23 hours
[4]Exfoliation	Water/PBS	24 hours
[4]Intercalation	Water/PBS	15 hours
[5]Hydrothermal method	Water	24 hours
[6]Hydrothermal-assisted ball milling	Water	3 days
[6]Hydrothermal-assisted ball milling	Acetone	>3 days
Our study	EG	>7 days

Table S3. Surface free energy of ethanol-water mixtures[7,8]

Ethanol vol%	0	5	10	45	75	100
γ/(mJ/m^2) at 20°C	72.8	56.4	48.1	32.6	29.8	23.7

Section S1. Determination of the effective surface hydroxyl density on M-2H-MoS_2 nanosheets.

The effective surface hydroxyl site density ($N_{s,eff}$) was determined using acid-base titration and Gran function analysis[9]. M-2H-MoS_2 nanosheets (30.0 mg) were dispersed in 30.0 mL water (1.0 g/L) under magnetic stirring in an N_2 atmosphere. The pH was adjusted to 4.50 with 1.0 M HCl, and then backtitrated to pH 11.0 with 0.01 M NaOH. After each NaOH addition, pH and cumulative NaOH volume were recorded when the pH change was less than 0.02 units in 5 minutes. A blank titration of the supernatant (dispersion after centrifugation at 8000 rpm for 10 min) was also performed for control. The titration data were analyzed using two Gran functions to estimate the $N_{s,eff}$ value. The two Gran functions, G_a and G_b, can be expressed as

$$G_a = (V_{SUS} + V)10^{-pH} \quad \text{(for the acidic side)} \tag{1}$$

$$G_b = (V_{SUS} + V)10^{pH-14} \quad \text{(for the alkaline side)} \tag{2}$$

where V_{sus} is the initial volume of the dispersion (L), and V is the cumulative volume of NaOH added (L). Gran function plots G_a and G_b against V yield two lines, which intersect the V-axis at V_{eq1} and V_{eq2}, respectively. The difference between these two volumes (V_{eq2}-V_{eq1}) represents the volume of NaOH required to completely deprotonate the protonated hydroxyl groups on the M-2H-MoS_2 surfaces.

$$N_{s,eff} = \frac{[(V_{eq2}-V_{eq1})_{Sample}-(V_{eq2}-V_{eq1})_{Control}]C_{NaOH}}{2m} \tag{3}$$

Where C_{NaOH} is the concentration of NaOH solution (mmol/L) and m is the used amount of M-2H-MoS_2 nanosheets (g).

References:

(1) Shen, J.; He, Y.; Wu, J.; Gao, C.; Keyshar, K.; Zhang, X.; Yang, Y.; Ye, M.; Vajtai, R.; Lou, J.; Ajayan, P. M. Liquid Phase Exfoliation of Two-Dimensional Materials by Directly Probing and Matching Surface Tension Components. *Nano Lett.* **2015**, 15 (8), 5449–5454. https://doi.org/10.1021/acs.nanolett.5b01842.

(2) Coleman, J. N.; Lotya, M.; O'Neill, A.; Bergin, S. D.; King, P. J.; Khan, U.; Young, K.; Gaucher, A.; De, S.; Ronan J. Smith, R. J.; Igor V. Shvets, I. V.; Arora, S. K.; Stanton, G.; Kim, H.; Lee, K.; Kim, G. T. Duesberg, G. S.; Hallam, T.; Boland, J. J.; Wang, J. J.; Donegan, J. F.; Grunlan, J.C. Moriarty, G.; Shmeliov, A.; Nicholls, R. J. N.; Perkins, J. M.; Grieveson, E. M.; Theuwissen, K.; McComb, D. W.; Nellist, P. D.; and Nicolosi, V. Two-Dimensional Nanosheets Produced by Liquid Exfoliation of Layered Materials. *Science*. **2011**, 331, 568-571. https://www.science.org/doi/10.1126/science.1194975.

(3) Forsberg, V.; Zhang, R.; Bäckström, J.; Dahlström, C.; Andres, B.; Norgren, M.; ANDERSSON, M.; Hummelgård, M.; Olin, H. Exfoliated MoS_2 in Water without Additives. *PLoS ONE*. **2016**, 11(4), e0154522. https://doi.org/10.1371/journal.pone.0154522.

(4) Kurapati, R.; Muzi, L.; de Garibay, A. P. R.; Russier, J.; Voiry, D.; Vacchi, I. A.; Chhowalla, M.; Bianco, A. Enzymatic Biodegradability of Pristine and Functionalized Transition Metal Dichalcogenide MoS_2 Nanosheets. *Advanced Functional Materials* **2017**, 27 (7), 1605176. https://doi.org/10.1002/adfm.201605176.

(5) Bo, Z.; Cheng, X.; Yang, H.; Guo, X.; Yan, J.; Cen, K.; Han, Z.; Dai, L. Ultrathick MoS_2 Films with Exceptionally High Volumetric Capacitance. *Adv. Funct. Mater.* **2022**, 12 (11), 2103394. https://doi.org/10.1002/aenm.202103394.

(6) Ahmadi, M.; Zabihi, O.; Li, Q.; Fakhrhoseini, S. M.; Naebe, M. A Hydrothermal-Assisted Ball Milling Approach for Scalable Production of High-Quality Functionalized MoS_2 Nanosheets for Polymer Nanocomposites. *Nanomaterials* **2019**, 9 (10), 1400. https://doi.org/10.3390/nano9101400.

(7) Sharma, M.; Roy, P. K.; Barman, J.; Khare, K. Mobility of Aqueous and Binary Mixture Drops on Lubricating Fluid-Coated Slippery Surfaces. *Langmuir* **2019**, 35 (24), 7672–7679. https://doi.org/10.1021/acs.langmuir.9b00483.

(8) Vazquez, G.; Alvarez, E.; Navaza, J. M. Surface Tension of Alcohol Water + Water from 20 to 50 .Degree.C. *J. Chem. Eng. Data* **1995**, 40 (3), 611–614. https://doi.org/10.1021/je00019a016.
(9) Jolsterå, R.; Gunneriusson, L.; Forsling, W. Adsorption and Surface Complex Modeling of Silicates on Maghemite in Aqueous Suspensions. *J. Colloid Interface Sci.* **2010**, 342 (2), 493–498. https://doi.org/10.1016/j.jcis.2009.10.080.
